# Genetic diversity and environmental adaptation in Ethiopian tef

**DOI:** 10.1093/g3journal/jkae303

**Published:** 2025-01-24

**Authors:** Kirsten Hein, Dejene Girma, John McKay

**Affiliations:** Department of Soil and Crop Sciences, Colorado State University, Fort Collins, CO 80523, USA; Graduate Degree Program in Ecology, Colorado State University, Fort Collins, CO 80523, USA; Ethiopian Institute of Agricultural Research, Addis Ababa 1000, Ethiopia; Department of Soil and Crop Sciences, Colorado State University, Fort Collins, CO 80523, USA; Graduate Degree Program in Ecology, Colorado State University, Fort Collins, CO 80523, USA

**Keywords:** landscape genomics, local adaptation, *Eragrostis tef*, redundancy analysis, genome–environment association, whole-genome resequencing

## Abstract

Orphan crops serve as essential resources for both nutrition and income in local communities and offer potential solutions to the challenges of food security and climate vulnerability. Tef [*Eragrostis tef* (Zucc.)], a small-grained allotetraploid, C_4_ cereal mainly cultivated in Ethiopia, stands out for its adaptability to marginal conditions and high nutritional value, which holds both local and global promise. Despite its significance, tef is considered an orphan crop due to limited genetic improvement efforts, reliance on subsistence farming, and its nutritional, economic, and cultural importance. Although pre-Semitic inhabitants of Ethiopia have cultivated tef for millennia (4000–1000 BCE), the genetic and environmental drivers of local adaptation remain poorly understood. To address this, we resequenced a diverse collection of traditional tef varieties to investigate their genetic structure and identify genomic regions under environmental selection using redundancy analysis, complemented by differentiation-based methods. We identified 145 loci associated with abiotic environmental factors, with minimal geographic influence observed in the genetic structure of the sample population. Overall, this work contributes to the broader understanding of local adaptation and its genetic basis in tef, providing insights that support efforts to develop elite germplasms with improved environmental resilience.

## Teaser

In this study, Hein *et al*. resequenced a georeferenced collection of *Eragrostis* tef to investigate genome-wide patterns of population structure and selection along abiotic environmental gradients across diverse cultivation ranges. Using genotype-environment association methods and differentiation-based scans, the authors identified key genomic regions and candidate genes under selection for climate adaptation. These findings were used to estimate adaptive genomic offset between current and projected climates, highlighting northern Ethiopia as a climate-vulnerable region under future scenarios. This paper targets readers in population and landscape genetics, integrating advanced genomic technologies and statistical approaches to uncover the genetic basis of adaptation in Ethiopian tef.

## Introduction

Divergent selection pressures across a species range naturally drive adaptive differentiation among populations, leading to the maintenance of genomic and phenotypic variation that underlies local adaptation. Such local adaptation is expected and observed in natural populations, traditional crop varieties, and its wild relatives, serving as a source for novel traits, alleles, and donor lines for developing plant varieties that thrive in current and future climates. Understanding the genetics of such adaptation and characterizing the selective agents that drive adaptive evolution allows a mechanistic approach to designing and predicting crop suitability across current and future target populations and environments for a given crop.

Recent studies have described the genetic architecture of adaptation and identified environmental-associated loci by conducting extensive genome-wide analyses of wild and domesticated species, e.g. Arabidopsis [*Arabidopsis thaliana* (L.) Heynh.] (Fournier-Level *et al*. 2011; Hancock *et al*. 2011; Lasky *et al*. 2012; Agren *et al*. 2013), rice [*Oryza sativa* (L.)] (Huang *et al*. 2010), maize [*Zea mays* (L.)] (Hufford *et al*. 2012; Josephs *et al*. 2019), sorghum [*Sorghum bicolor* (L.) Moench] (Morris *et al*. 2013; Lasky *et al*. 2015), soybean [*Glycine soja* (Sieb. & Zucc.)] (Wang *et al*. 2022), barley [*Hordeum vulgare* (L.)] (Abebe *et al*. 2015), pine [*Pinus taeda* (l.)] (Eckert *et al*. 2010a; Eckert *et al*. 2010b; Lu *et al*. 2019), and peach [*Prunus persica* (L.)] (Li *et al*. 2021). Outlier detection methods are widely used to identify genomic regions under positive selection, providing critical insights into the genetic basis of adaptation, as reviewed by Biwas and Akey (2006) and Saravanan *et al*. (2021). Research on crop species, especially in traditional varieties, has shown their ability to adapt to diverse climates over generations of natural and artificial selection. However, in many crops, the significant reduction in effective population sizes during both domestication and crop improvement has led to the loss of adaptive variation and the accumulation of deleterious mutations (Moyers *et al*. 2018). In annual crops, there has been an observed loss of ∼40% of diversity compared with wild relatives (Miller and Gross 2011). Traditional varieties and their wild relatives offer abundant genetic diversity, making them invaluable assets for breeding programs (Bolger *et al*. 2014; Li *et al*. 2018; Gao *et al*. 2019; Wang *et al*. 2022).

Tef [*Eragrostis tef* (Zucc.)] is a small-grained C_4_ cereal crop that is of significant importance for smallholder farmers in Eastern Africa. Belonging to the Poaceae family and genus *Eragrostis*, tef is a self-pollinated annual plant that is cultivated primarily as a grain for human consumption or as biomass for forage. As a short-day plant, tef is well adapted to altitudes ranging from 1,800 to 3,000 m, with optimal growth occurs at mean temperatures of 15–21°C, annual rainfall between 400 and 750 mm, and in fertile soils with a pH range of 5.2–5.5 (Belay and Assen 2023). Tef is of major cultural and economic importance in Ethiopia, where the grain is milled to produce injera, a nutrient-dense and gluten-free Ethiopian bread. In Western countries, tef is valued as a healthy food alternative for individuals with Celiac disease. Despite its high nutritional value and ability to thrive under unfavorable conditions compared with other locally grown cereals, tef is considered an orphan crop and has experienced limited genetic improvement.

In the last decade, genomics research in tef has gained traction with the release of draft genome sequences (Cannarozzi *et al*. 2014) and a high-quality genome sequence (VanBuren *et al*. 2020). Tef has an allotetraploid origin and 20 chromosome pairs with a relatively small genome of ∼622 Mb (VanBuren *et al*. 2020). Studies investigating phenotypic variation in tef germplasm have found differences in days to maturity, harvest index, number of culm nodes, and diameters of first and second shoot culm internodes across collections from different elevations and regions (Tadesse 1993; Assefa *et al*. 2000, 2002; Kefyalew *et al*. 2000; Woldeyohannes *et al*. 2020). Advancements in breeding techniques, complemented by insights into the diversity and inheritance of target agronomic traits, have been key to the genetic improvement of tef and have contributed to the total release of 49 improved varieties from the Ethiopian Institute for Agricultural Research since the 1950s (Assefa *et al*. 2011; Ministry of Agriculture and Livestock Resources 2019).

Ethiopia is one of the most agroecologically diverse countries in the world. As 1 of the 12 Vavilovian centers of diversity, it features a vast range of altitudinal clines spanning from 110 to 4,620 m above sea level (Vavilov 1951; Abebe *et al*. 2015). These regions experience large variability in both seasonal and annual climatic conditions. Although environmental heterogeneity may be a major driver of the richness of plant biodiversity across Ethiopia, climate change is predicted to lead to a major decrease in crop productivity through increased water and heat stress, the prevalence of diseases and pests, and shortened growing seasons (IPCC 2014). To meet current and future agricultural demands across diverse agroecologies, it is essential to survey the existing allelic diversity of traditional crop varieties grown across their range of cultivation.

In this study, we tested the hypothesis that these traditional tef varieties are locally adapted along an abiotic environmental gradient throughout its range of cultivation. To address this hypothesis, we sampled and resequenced a geographically diverse collection of tef germplasm to (1) characterize the extent and geographic distribution of the population structure; (2) identify genetic variations associated with multivariate environmental gradients; (3) isolate environmental factors contributing to population differentiation; (4) detect genomic regions that have undergone recent selective sweeps; and (5) predict the suitability of existing germplasm to future climates. To achieve these aims, we employed multivariate genotype–environment association methods and differentiation-based genome scans.

## Materials and methods

### Samples, variant calling, and genotyping

The tef germplasm panel used in this study was sourced from the Ethiopian Biodiversity Institute as part of a collaborative project with the Ethiopian Institute of Agricultural Research (EIAR). The panel consisted of 96 traditional tef varieties, each with known geographical coordinates from across major production regions. A complete list of the germplasm collection is provided in Supplementary Table 1.1 in Supplementary File 1. Seeds from this collection were sown in a greenhouse at the EIAR in Ethiopia. After 4 weeks, young leaves were sampled from of single plants representative of each variety. Genomic DNA was extracted from the plant leaf tissue in 96-well plate format using a modified version of the cetyltrimethylammonium bromide method (Chua *et al*. 1990). DNA concentration and quality were determined using a 1.5% agarose gel and a Nano Drop Spectrophotometer. The extracted DNA was sent to the BioFrontiers Sequencing Core at the University of Colorado Boulder (Boulder, CO, USA) for unique dual indexing of 10-bp libraries. Genomic libraries were then sent to the Genomics and Microarray Core at the University of Colorado Anschutz Medical Campus (Aurora, CO, USA) for paired-end whole-genome sequencing (2 × 150 bp at ∼10× coverage) using an Illumina NovaSeq 6000 platform.

The raw paired-end 150-nt reads were scanned for adapter sequences, low-quality base pairs, and reads shorter than 35 bp were trimmed using Fastp version 0.19.6 (Chen *et al*. 2018). Trimmed reads were aligned to the *E. tef* v3 genome (CoGe id50954) (VanBuren *et al*. 2020) using BWA-MEM version 0.7.17 with default settings (Li 2013). The resulting Sequence Alignment Map files were sorted by chromosome, annotated for read group information, filtered for improperly paired reads, and reads with a mapping quality of <10, were converted to Binary Alignment Map format and indexed using SAMtools version 1.1 (Li *et al*. 2009) before proceeding to single nucleotide polymorphisms (SNPs) calling using GATK Best Practices recommendations (Poplin *et al*. 2017; van der Auwera and O’Connor 2020). SNP calling was performed using GATK HaplotypeCaller with default settings, and the resulting genomic variant call format (VCF) files were combined into a single database using GenomicDBImport for joint genotyping with GenotypeGVCF (McKenna *et al*. 2010). The resulting VCF files were filtered to contain only bi-allelic SNPs that met quality criteria using R/vcfR (Knaus and Grünwald 2017) and GATK SelectVariants and VariantFiltration tools (McKenna *et al*. 2010). Finally, TASSEL version 5.2.83 (Bradbury *et al*. 2007) was utilized to remove loci with a minor allele frequency ≤5% and heterozygous genotype calls ≥50%, as well as taxa that possessed a genotyping rate ≤80%. The filtered VCF file comprised 538,252 markers from 87 individuals and was used for subsequent analyses.

### Climate data

Statistical analyses of climatic variation were conducted on 87 georeferenced traditional tef varieties from 75 locations to evaluate the environmental conditions across sampling sites. Bioclimatic (bio1–bio19) and altitudinal data were obtained through the latitude and longitude coordinates from each site (see Supplementary Table 1.2 in Supplementary File 1) using WorldClim version 2.1 layers at 10-min resolution based on long-term averages for 1970–2000 (Fick and Hijmans 2017). Descriptions and calculations for each WorldClim bioclimatic variable are presented in Supplementary Table 1. The relationships among the 20 environmental variables across 75 locations were examined through principal component analysis (PCA) using R/FactoMineR (Lê *et al*. 2008) and R/factoextra (Kassambara and Mundt 2020).

Future climate scenarios were projected using the Coupled Model Intercomparison Project (CMIP6) and shared socioeconomic pathways (SSPs) developed by the IPCC. The latest phase of this model integrates new emission and land-use scenarios compared with previous models (Eyring *et al*. 2016; O’Neill *et al*. 2016; Stouffer *et al*. 2017; Wyser *et al*. 2020). Our study focused on the WorldClim v2.1 bias-corrected SSP2-4.5 scenario, which is considered a median pathway among the 5 SSP scenarios (SSP1–SSP5) that represent varying mitigation and adaptation challenges (Huang *et al*. 2019). Using a set of 12 CMIP6 global circulation models (listed in Supplementary Table 2), we calculated the mean ensemble of projected climate change for the period 2061–2080 implementing R/raster (Boucher *et al*. 2019; Dix *et al*. 2019; EC-Earth 2019; Good 2019; Good *et al*. 2019; Hijmans 2023; Lovato *et al*. 2023; NASA Goddard Institute for Space Studies 2020; Schupfner *et al*. 2019; Shim *et al*. 2020; Shiogama *et al*. 2019; Song *et al*. 2019; Volodin *et al*. 2019; Yukimoto *et al*. 2019).

### Inference of population structure

Accounting for population structure in genotype-to-phenotype association studies is essential for reducing the number of false positives (Pritchard *et al*. 2000; Yu *et al*. 2006; Excoffier and Ray 2008; Kang *et al*. 2008; Coop *et al*. 2009; Excoffier *et al*. 2009; Rellstab *et al*. 2015). PCA and genome-wide discriminant analysis of principal components (DAPC) were conducted to quantify population structure using R/adegenet (Jombart 2008; Jombart and Ahmed 2011). Individual genetic ancestries were estimated using the Q matrix to determine the appropriate number of genetic clusters (*K*) through cross-validation. *K* values for subpopulations ranging from *K* = 1 to *K* = –10 were tested, and *K* = 5 was selected for analyses. To estimate the degree to which marker data were structured by each of the environmental predictors, we examined the relationships between the ordinal axes generated by the genome-wide DAPC and the environmental predictors. The statistical dependence of each ordinal axis on each predictor was assessed using the corr.test function in R/psych (Revelle 2023), with the method set to “spearman” and α level set to 0.05.

To assess the extent of isolation by distance (IBD) and isolation by environment (IBE) due to population structure, we implemented the Mantel test using the Mantel function in R/vegan based on Spearman's rank correlation. Spatial autocorrelation was performed along latitude and longitude using spherical coordinates to create a geographical distance matrix (in km) for sampling locations with 2 or more collected varieties, using the rdist.earth function in R/fields (Nychka *et al*. 2021). Similarly, environmental autocorrelation was carried out for these locations using Euclidean pairwise distances across 20 environmental variables, calculated using R/rdist (Blaser 2020). Site-level genetic distances were estimated as pairwise fixation indices (*F_ST_*) using the genet.dist function in R/hierfstat (Goudet and Jombart 2022), applying the “WC84” (Weir and Cockerham 1984) method. Although our panel consisted of 96 individuals sampled from 75 distinct geographic locations, only 16 locations had more than 1 sample. As a result, the distance matrices for the IBD and IBE analyses were based on 39 individuals from these 16 locations. Negative *F_ST_* estimates were converted to zero values for subsequent analyses. The significance of each Mantel statistic (*r*) was evaluated with a two-tailed 95% confidence interval centered on the null hypothesis of no spatial or environmental correlation with genetic structure (*r* = 0) with 9,999 permutations.

### Multivariate genotype × environment association analysis

Redundancy analysis (RDA), a constrained ordination technique, was employed to detect adaptive processes and multilocus architectures associated with environmental variations using R/vegan (Oksanen *et al*. 2022). As a common multivariate genotype–environment association approach for exploring the relationship between genetic and environmental data, RDA has demonstrated promise for identifying sets of covarying loci that most strongly correlate with environmental predictors under varying selective strengths (Lasky *et al*. 2012; Legendre and Legendre 2012; Bourret *et al*. 2013, 2014; Rellstab *et al*. 2015; Forester *et al*. 2018). As RDA requires complete data frames, missing values identified within the genotype matrix (∼14.38% of the data) were imputed by using the most common genotype at each marker across all individuals before converting the data to allele counts for each locus and variety (Forester *et al*. 2018). Moreover, RDA is highly susceptible to problems when using highly correlated predictors. The use of highly correlated predictors in the RDA can increase its susceptibility to multicollinearity, thereby hindering the accurate assessment of individual predictor contributions (Dormann *et al*. 2013). To address this issue, we identified correlated environmental predictors of |*r*| > 0.70 using R/psych and excluded them from RDA model testing. Multicollinearity was further controlled by checking the variance inflation factors, with a threshold of 5 (Zuur *et al*. 2010).

Variance partitioning quantifies the proportion of variance attributed to a specific set of explanatory variables (e.g. climate) after accounting for the effects of other variables (e.g. population structure) (Legendre and Legendre 2012). We used partial RDA-based variance partitioning to separate and evaluate the contributions of climate and population structure in explaining genetic variation. Formal tests for the analysis of variance (ANOVA) were employed to assess the significance of all model combinations. Each model's statistical significance was evaluated using a permutation-based with 999 permutations and a significance level (α) of 0.05 (Legendre and Legendre 2012). The null hypothesis is that there is no linear relationship between genetic data and environmental predictors. The number of constrained axes retained for loci detection was visually determined using a scree plot of the canonical eigenvalues. We then identified the covariates that were most strongly correlated with each candidate locus (i.e. highest correlation coefficient) to group candidates by potential driving environmental variables. Loci loading in the tails on each RDA axis, with a two-tailed 99.9% confidence interval around the null hypothesis (±3.29 SD from the mean axis loading; *P* < 0.001), were identified as putative adaptive loci (Forester *et al*. 2018; Capblancq and Forester 2021).

### Identification of selection signatures

We implemented 2 alternative approaches that incorporate intrapopulation (iHH12) and interpopulation (${\bar{F}}_{ST}$) statistics.

Integrated haplotype homozygosity pooled (iHH12) is a linkage disequilibrium (LD)-based method that can identify genomic regions under positive selection by measuring the extent to which haplotypes are shared and homozygous in a population (Maynard Smith and Haigh 1974; Voight *et al*. 2006). For each site, we calculated the iHH12 score using the software selscan v2.0.0 (Szpiech and Hernandez 2014), which offers better power than the integrated haplotype score for soft sweep detection (Garud *et al*. 2015; Torres *et al*. 2018). These iHH12 scores were then normalized to *Z*-scores (standard normal scores), and 20-kb sliding windows were employed to identify potential target regions of positive selection, considering only the top 1% of the distribution of iHH12 values. By treating all samples from different locations as 1 population, we can identify genomic regions where recent selective sweeps have occurred without considering specific environmental factors. This approach can reveal general selection patterns that are advantageous across various environments, highlighting coordinated changes in allele frequency common to all tested tef varieties.

The concept of ${\bar{F}}_{ST}$, pioneered by Wright (1949), assesses differences in allele frequencies across populations and can be used to identify genomic regions with distinct fixation patterns. ${\bar{F}}_{ST}$ values can range from 0, which suggests no genetic differentiation, to 1, complete genetic differentiation. Mean ${\bar{F}}_{ST}$ was estimated using a sliding window approach weighted by the number of sites, with a window size of 20-kb and a step size of 20-kb, using the software VCFtools version 0.1.16 (Weir and Cockerham 1984; Danecek *et al*. 2011). To examine the degree of divergent selection within the distribution of select environmental variables, individuals were grouped based on the lower and upper quartiles of each explanatory variable used in the RDA. Altitude (in meter above sea level) was included as an additional variable in the analysis due to the considerable variation in adaptive and farmer-preferred traits within and across altitudinal classes (Woldeyohannes *et al*. 2020). Genomic windows with ${\bar{F}}_{ST}$ values ≤0 were omitted from the analysis. The genomic windows with the highest 1% ${\bar{F}}_{ST}$ values were considered to represent signatures of divergent selection across environmental gradients. iHH12 scores and sliding window ${\bar{F}}_{ST}$ were visualized using R/ggplot2 (Wickham 2016) and R/qqman (Turner 2018), respectively.

### Functional annotation and candidate gene analysis

Gene candidates were identified as gene IDs in the *E. tef* v3 genome located within 1-kb downstream or upstream of significant RDA loci. For the functional annotation of these gene models, we performed a blastp search against the National Center for Biotechnology Information (NCBI) nonredundant protein database combined with the gene ontology (GO) annotation database of genes based on putative rice orthologs (see Yu *et al*. 2023). The top homologous matches in the NCBI query were selected based on maximum sequence similarity (≥95%), maximum bit-score (≥50), and an *E*-value cutoff of 0.05. GO enrichment analysis of significant RDA loci was performed using R/clusterProfiler (Yu *et al*. 2012; Wu *et al*. 2021) following the methodology outlined by Yu *et al*. (2023). Furthermore, gene models were evaluated for the presence of locus-specific nonsynonymous sequence changes and evidence of overlap with regions under positive selection.

### Predicting adaptive landscapes and local maladaptation

Following the methodologies described by Capblancq and Forester (2021) and Steane *et al*. (2014), we identified regions within the landscape where the adaptive allele frequencies in local gene pools align with the inferred fitness optima associated with local climates. Leveraging the genotype–environment relationship identified through RDA, we employed these candidate adaptive markers and their prospective environmental drivers to predict an index of genetic composition. This process involved employing loci as multivariate responses in an “adaptively enriched” RDA, where bioclimatic variables were explanatory factors. Subsequently, by using environmental variable scores along the first 2 RDA axes, we calculated a genetic-based adaptive index for each environmental pixel using the following formula:


$$\mathrm{Adaptive}\;\mathrm{index}=\;\overset{n}{\underset{i=1}{\sum }}\;a_{i}b_{i}$$

Here, “*a*” represents the loading score of the bioclimatic variable along the RDA axis, “*b*” denotes the standardized value specific to that variable at the focal pixel, and “*i*” pertains to 1 of the *n* variables used in the RDA model. These climatic variable scores were calculated based on how each predictor influences adaptive genetic variation in terms of magnitude and direction along the RDA axes. Consequently, the adaptive index serves as an estimate of genetic similarity or dissimilarity among all pixels across the landscape considering the values of bioclimatic predictors at each location (Steane *et al*. 2014; Capblancq and Forester 2021).

To predict future maladaptation, adaptive indices were calculated for each environmental pixel across the Ethiopian landscape considering both current and future environmental conditions. The contrast between these predictions indicates the necessary shift in adaptive index to accommodate projected climates. Alternatively termed genetic/genomic offset (Fitzpatrick and Keller 2015), risk of nonadaptedness (Rellstab *et al*. 2016), or genomic vulnerability (Bay *et al*. 2018), this proxy was estimated using the CMIP6 SSP2-4.5 projected climate changes for the period 2061–2080. The projections were then mapped across Ethiopia to identify areas potentially at risk of maladaptation.

## Results

### Limited geographic influence on genetic diversity in sample collection of traditional tef varieties

**Fig. 1.**
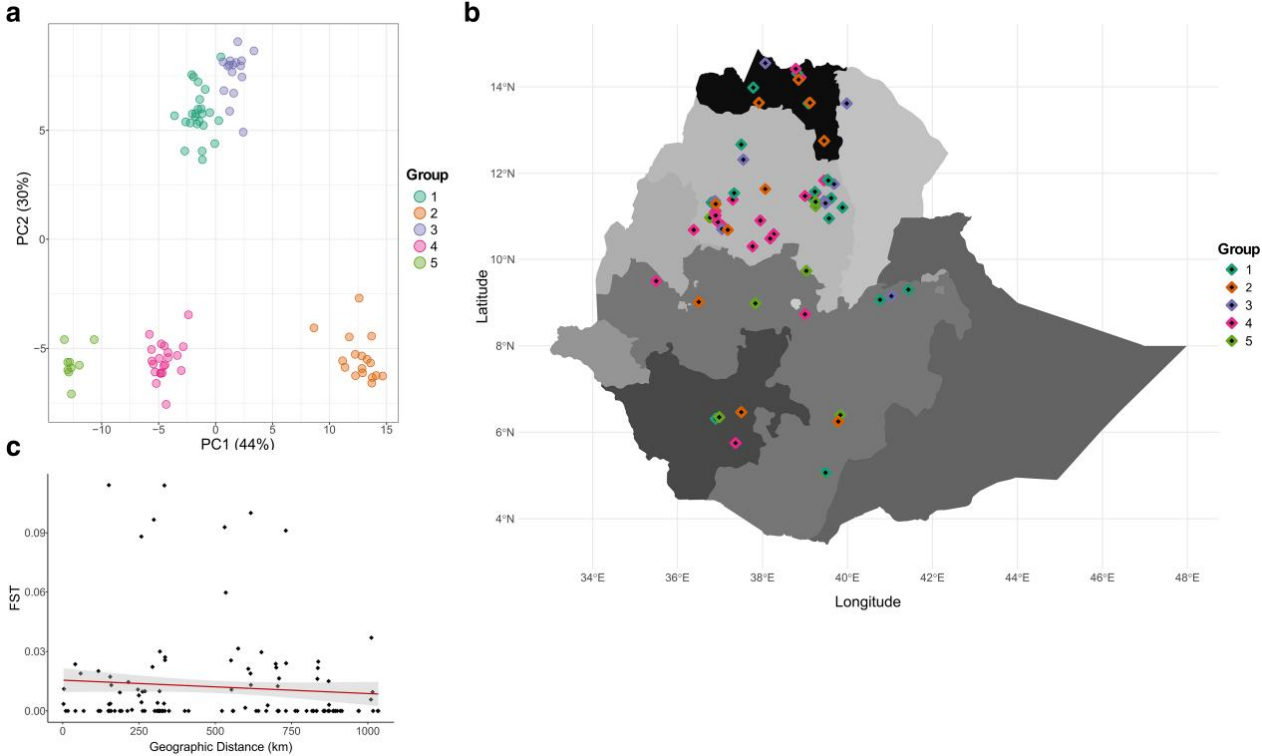
Geographic distribution and population structure of the 87 traditional tef varieties sampled across Ethiopia. a) DAPC using genome-wide markers. Taxa are color-coded based on their respective DAPC genetic clusters, as defined in the legend. b) Sampling distribution of tef varieties across Ethiopia, color-coded according to respective DAPC genetic clusters. c) Linear regression of ${\bar{F}}_{ST}$ values as a function of geographic distance between locations with 2 or more varieties.

A total of 538,252 markers were used to infer the population structure of 87 traditional tef varieties. Genome-wide DAPC and IBD across individuals identified high levels of gene flow across geography, suggesting a lack of spatial influence on tef genetic diversity. The *K*-means clustering algorithm identified *K* = 5 as the optimal number of clusters and grouped individuals based on their genetic similarity, with ∼74% of the genetic variation explained by the first 2 principal components (Fig. 1a; see Supplementary Table 1.3 in Supplementary File 1). Cluster 1 consisted of 24 individuals, cluster 2 consisted of 17 individuals, cluster 3 consisted of 16 individuals, cluster 4 consisted of 21 individuals, and cluster 5 consisted of 9 individuals. Genetic clustering was based on an algorithm that minimizes variation within clusters while maximizing variation among clusters, which indicates a recent shared evolutionary history among individuals within clusters. Interestingly, genetically similar individuals did not cluster by geography (Fig. 1b). Furthermore, there were no significant differences in environmental and geographical distribution among clusters. The statistical dependence of each DAPC axis on environmental predictors were weak, with LD1 and LD2 showing the highest correlation to mean temperature in the wettest quarter (LD1: *r* = 0.224, *P* = 0.037; LD2: *r* = 0.280, *P* = 0.009). Finally, we tested for spatial and environmental autocorrelation and found no evidence of IBD or IBE, where *F_ST_* values were not significantly correlated to geographic distance (Mantel *r* = −0.006, *P* = 0.505) or environmental distance (Mantel *r* = 0.162, *P* = 0.071) (Fig. 1c).

### Low proportion of confounded genetic variance shared between climate and population structure

PCA biplots were created to explore the relationships among 20 bioclimatic variables across 75 locations (Supplementary Fig. 1). The first 3 principal components accounted for 81.70% of the total variance in the data. These biplots highlight a clear separation between temperature variables (43.20% variance explained), seasonality variables (26.50%), and precipitation variables (12%). After removing highly correlated predictors, 6 of the 20 bioclimatic variables were selected for RDA based on model significance (*P* = 0.031) and representation across the predictor types. This included the annual mean temperature (bio1), temperature annual range (bio7), precipitation of the wettest month (bio13), precipitation of the driest month (bio14), precipitation of the warmest quarter (bio18), and precipitation of the coldest quarter (bio19). A variance inflation factor check indicated insignificant multicollinearity among the 6 selected predictors (all values <5).

**Fig. 2.**
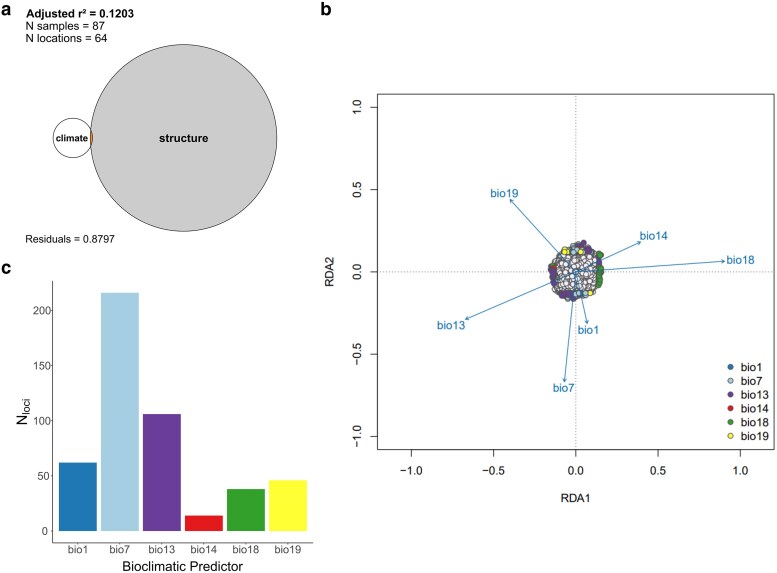
Genotype–environment association using RDA. a) Variance partitioning of genomic diversity explained by a combination of the bioclimatic variables “climate” and PC scores “structure,” represented as an Euler plot. b) RDA biplot illustrating the relative contribution of climate shaping the genetic structure of 538,252 markers across 87 traditional tef varieties, conditioned on 6 bioclimatic predictors (bio1, annual mean temperature; bio7, temperature annual range; bio13, precipitation of the wettest month; bio14, precipitation of the driest month; bio18, precipitation of the warmest quarter; bio19, precipitation of the coldest quarter). The outlier loci were grouped by color, reflecting their highest correlation with specific predictors. c) Variation in the counts of significant RDA loci (*N* = 482) that were attributed to different bioclimatic predictors. The bars are color-coded according to the legend in b).

Partial RDA-based variance partitioning was performed to disentangle the effects of climate and population structure on genetic variation. We used 2 sets of explanatory variables: (1) 6 bioclimatic variables (“climate”) and (2) 2 proxies for genetic structure (population scores from the first 2 axes of a genetic PCA on 538,252 markers, termed “structure”; see Supplementary Table 1.3 in Supplementary File 1). Six models were analyzed, with individual allele counts as the response variable (Table 1). Together, climate and population structure significantly explained 12.03% (*P* ≤ 0.05) of the total genetic variance across tef varieties. Climate alone accounted for 0.45% of total genetic variation. Variance partitioning indicated that a low proportion of confounded variance shared between climate and population structure explained 0.18% of the total genetic variation (Fig. 2a; Table 1). This reflects a low degree of collinearity between the sets of explanatory variables. In this study, we evaluated RDA models both with and without controlling for population structure to minimize false negatives. Due to low spatial structure and collinearity between genetic structure and climate, we focused on the pure climate model (i.e. without population structure-based corrections) on downstream analyses. The results obtained from both RDA models are provided in Supplementary Tables 1.5 and 1.6 in Supplementary File 1.

**Table 1.** Effect of climate and genetic structure on genetic variation across RDA models.

*RDA models*		*Df*	*R^2^*	*Adjusted R^2^*	*P(>F)*
*Full model*	G ∼ climate + structure	8	0.202	0.120	0.001^a^
*Pure climate*	G ∼ climate	6	0.075	0.006	0.049^b^
*Pure structure*	G ∼ structure	2	0.115	0.115	0.001^a^
*Partial climate*	G ∼ climate | structure	6		0.004	0.028^b^
*Partial structure*	G ∼ structure | climate	2		0.114	0.001^a^
*Confounded climate/structure*		0		0.002	
*Residual*				*0.880*	

^a^ *P* ≤ 0.001; ^b^*P* ≤ 0.05. G, genetic variation; climate, dataset of 6 bioclimate variables selected for downstream analyses.

### RDA identifies loci associated with multivariate environmental gradients

The constrained ordination of the pure climate model (hereafter referred to as “RDA model”) explained ∼0.63% of the 538,252 variants (*r*^2^ = 0.076; adjusted *r*^2^ = 0.006), consistent with the expectation that most loci are likely to be neutral and not strongly associated with environmental predictors (Table 1). We then assessed the optimal number of constrained axes for locus detection by constructing a scree plot of the canonical eigenvalues. The first 2 constrained axes captured the majority of the variation in the model, with RDA1 explaining 25.92% of the variation and RDA2 explaining 18.77%, resulting in a cumulative variance of 44.69%. Consequently, we selected the first 2 constrained axes for further investigation. By examining histograms of the loadings for each axis, we observed a relatively normal distribution. Loci with loadings centered around zero lack a significant relationship with environmental predictors, whereas loci with loadings in the tails of the distribution are more likely to be under selection by these predictors or other factors correlated with them (Supplementary Fig. 2). We identified loci in the tails of these distributions below a significance cutoff of 0.001. This resulted in 482 candidate outlier loci, with 63 loci for RDA1 and 419 loci for RDA2.

The majority of the 482 RDA loci (44.81%) exhibited the strongest correlations (*r*_max_ = 0.420) with the temperature annual range (bio7), followed by 12.86% of loci with the strongest correlations (*r*_max_ = 0.440) with the precipitation of the wettest month (bio13) (Fig. 2c). To investigate the relationships between the RDA loci and predictors in more detail, we plotted their loadings along RDA1 and RDA2 (Fig. 2b). The resulting RDA biplot demonstrates the correlation between genetic variation and environmental predictors, highlighting how each RDA axis varies along adaptive gradients. Loci strongly loading on RDA1 were associated with multilocus haplotypes associated with precipitation of the wettest month (bio13), precipitation of the driest month (bio14), and precipitation of the warmest month (bio18). In contrast, loci strongly loading on RDA2 represent haplotypes associated with annual mean temperature (bio1), temperature annual range (bio7), and precipitation of the coldest quarter (bio19). These findings highlight novel candidate regions and multilocus haplotypes to better understand tef adaptation across Ethiopia.

### High ${\bar{F}}_{ST}$ regions reveal patterns of genetic divergence across environmental distributions

Genome-wide analysis of genetic differentiation demonstrated a low to moderate level of genetic divergence across environmental variables, with average ${\bar{F}}_{ST}$ values of 0.047 ± 0.050 (Supplementary Fig. 3; see Supplementary Table 1.9 in Supplementary File 1). Among the environmental variables analyzed (annual mean temperature—bio1, temperature annual range—bio7, precipitation of the wettest month—bio13, precipitation of the driest month—bio14, precipitation of the warmest quarter—bio18, precipitation of the coldest quarter—bio19, and altitude), we identified 622 genomic regions (comprised of 99 unique regions) with ${\bar{F}}_{ST}$ values 0.281 ± 0.072, derived from the top 1% of the distribution (Supplementary Table 3). Our analysis revealed prominent genetic signals on chromosomes 1A (18,880,001–19,140,001 bp), 1B (31,000,001–31,180,000 bp), and 4B (13,600,000–13,940,000 bp) that were uniquely associated with differences in precipitation of the warmest quarter (bio18), annual mean temperature (bio1), and precipitation of the wettest month (bio13), respectively (Supplementary Fig. 3). Additionally, colocalized genetic signals were observed across multiple variables. Notably, associations between annual mean temperature (bio1), precipitation of the warmest quarter (bio18), and altitude were identified on chromosomes 3A (5,600,001–6,100,000 bp) and 7A (14,260,001–14,500,000 bp). Two regions on chromosome 5A (1,502,000–17,500,000 bp; 23,100,001–23,410,000 bp) were also shared between annual mean temperature (bio1), precipitation of the warmest quarter (bio18), and altitude, suggesting a shared genetic response to these environmental variables.

### Absence of colocalization between recent selective sweeps and climate-correlated loci

**Fig. 3.**
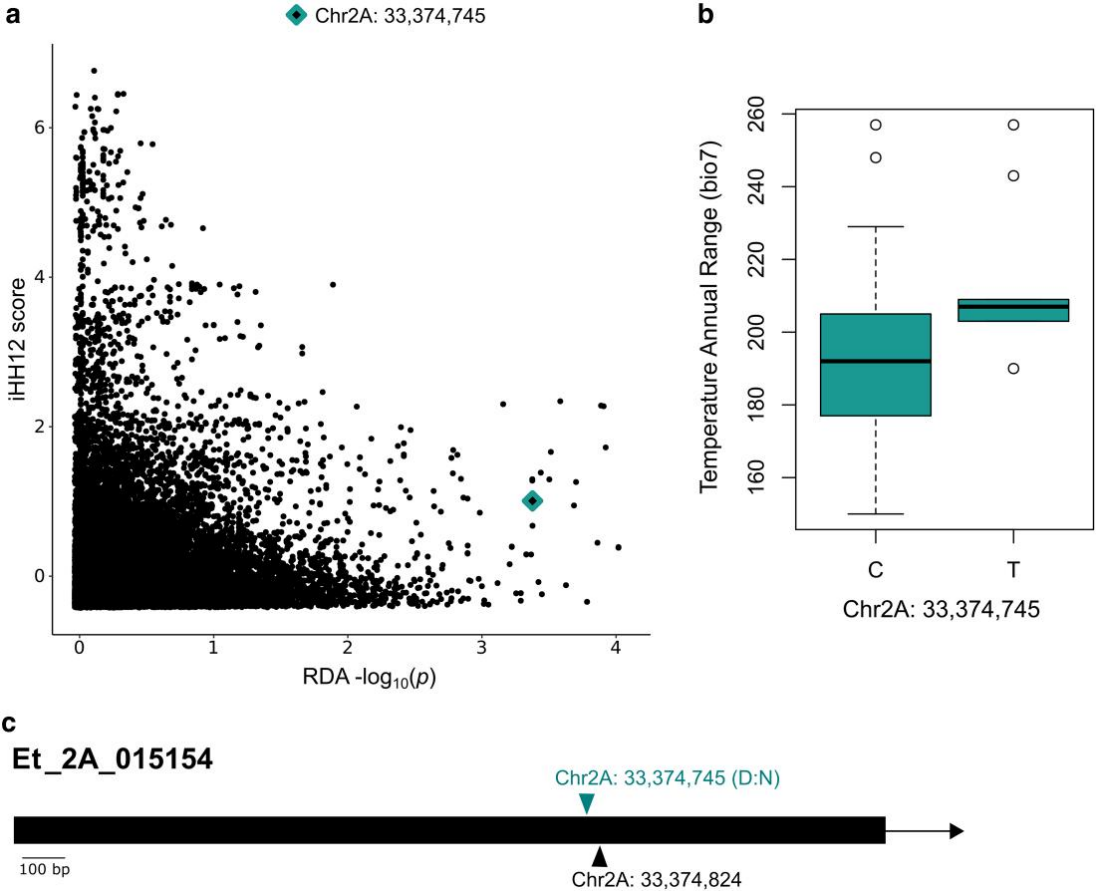
Characterization of candidate gene Et_2A_015154. a) Relationship between log-transformed *P*-values from the pure climate RDA model and iHH12 scores on chromosome 2A, with the locus associated with Et_2A_33374745 highlighted by a diamond shape. b) Variation in temperature annual range (bio7) based on allelic state at the locus conferring nonsynonymous nucleotide substitution at Et_2A_33374745. c) Gene model of Et_2A_015154 indicating the positions of 2 RDA-significant loci A nonsynonymous substitution is marked by a green triangle, and a synonymous substitution by a black triangle. Amino acid substitutions are represented by letter codes within the label.

**Fig. 4.**
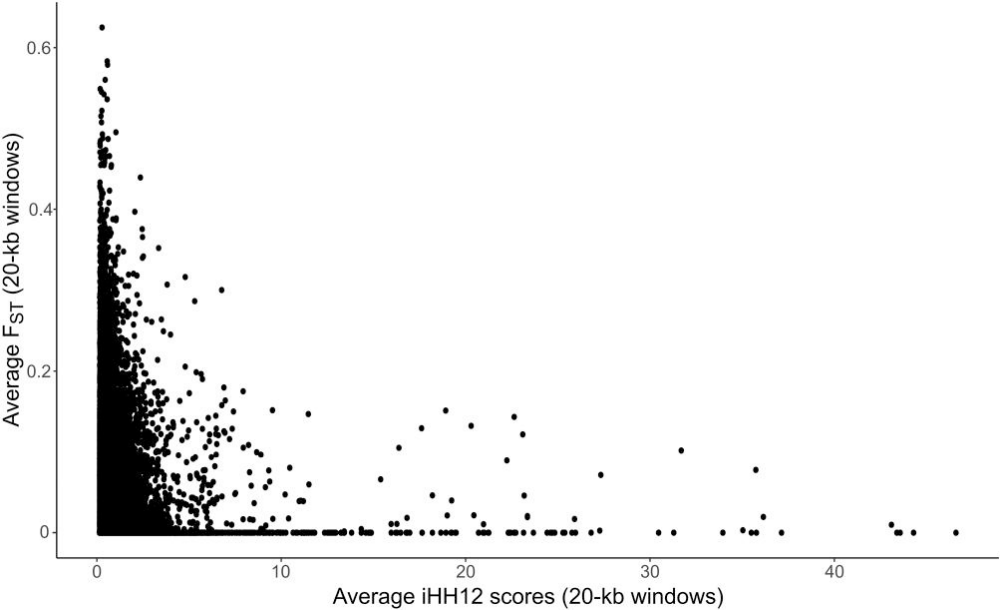
Genome-wide patterns of environmental divergence (${\bar{F}}_{ST}$) and iHH12 scores based on a subset of 100,000 loci sampled without replacement. ${\bar{F}}_{ST}$ values were derived from analyses of annual mean temperature (bio1), temperature annual range (bio7), precipitation of the wettest month (bio13), precipitation of the driest month (bio14), precipitation of the warmest quarter (bio18), precipitation of the coldest quarter (bio19), and altitude.

Genome-wide selection scans using the haplotype-based statistic iHH12 identified 192 genomic windows in the top 1% of iHH12 values, encompassing 5,137 loci and spanning 3.84-Mb across all 20 chromosomes (see Supplementary Table 1.8 in Supplementary File 1). Notably, chromosomes 1A, 1B, and 2A showed the highest enrichment for selective sweeps. These regions spanned from 1A (33,140,001–40,300,001 bp), 1B (33,140,001–35,480,001 bp), and 2A (28,760,001–35,420,001 bp), with peak signals at 1A:21,220,001, 1B:27,900,001, and 2A:33,220,001. However, these regions did not colocalize with climate-associated RDA loci. Further analysis revealed an inverse relationship between iHH12 scores and log-transformed *P*-values of the RDA loci. As shown in Fig. 3a, higher transformed RDA *P*-values were associated with lower iHH12 scores, suggesting that the climate-associated RDA loci have not undergone recent selective sweeps. Similarly, genome-wide comparisons between the iHH12 scores and aggregated ${\bar{F}}_{ST}$ values showed that highly divergent alleles were predominantly associated with lower iHH12 values (Fig. 4). This finding supports the predictions that more divergent alleles are maintained at intermediate frequencies, whereas loci associated with higher iHH12 scores occur at higher frequencies within a single common haplotype.

### GO enrichment analysis highlights 2 candidate genes associated with stress response

Among the 482 RDA loci, 145 (30.1%) were located within 1-kb upstream or downstream of *E. tef* v3 coding sequence regions, with 32 (6.6%) resulting in nonsynonymous nucleotide substitutions. Detailed genotype information and associated metadata for each nonsynonymous site identified by the RDA are provided in Supplementary Table 1.7 in Supplementary File 1. GO enrichment analyses of the 145 loci revealed 31 biological process GO terms linked to 92 genes (FDR; *P* < 0.05) across 5 bioclimatic variables (Supplementary Fig. 4; see Supplementary Tables 1.10 and 1.11 in Supplementary File 1). The annual mean temperature (bio1) was excluded from the analysis due to a lack of gene sets with associated gene models in the GO annotation database. We identified 5 functional categories represented by the top 19 enriched GO terms involving 38 genes. These categories included allantoin gene RNA, organelle component organization, protein stress conjugation, intracellular blue light, and cotyledon cytokinin stimulus (Supplementary Fig. 4). The GO functional categories exhibited minimal overlap among different bioclimatic variables, except for temperature annual range (bio7), precipitation of the wettest month (bio13), and precipitation of the warmest quarter (bio18), which shared terms in the allantoin gene RNA functional group. Moreover, the temperature annual range (bio7) and precipitation of the wettest month (bio13) shared the GO term for the cellular response to salt stress (GO:0071472). The largest cluster was related to stress responses and protein modification regulation, featuring 18 genes. Within this cluster, 3 RDA loci were identified with nonsynonymous changes in the coding sequences of Et_1B_010994: c.694G > C (p.Pro232Arg), Et_1B_010994: c.652C > G (p.Gly218Ala), and Et_1B_010993: c.301A > T (p.Glu99Val) (GO: 0071470; GO: 0071472).

We integrated the results of the RDA and ${\bar{F}}_{ST}$ analyses, which revealed 2 loci on chromosome 7A proximal to a genetic signal shared among 3 environmental variables: annual mean temperature (bio1), precipitation of the warmest quarter (bio18), and altitude. These loci, positioned at 14,652,594 and 14,764,385 bp, were ∼390 and 500 kb from the signal, respectively. Both loci showed significant positive correlations (*P* < 0.05) with the temperature annual range (bio7) in the RDA model (*r* = 0.300 and 0.268). Based on the v3 *E. tef* genome annotation, 1 locus was located within an intron of the gene model Et_7A_051114. An NCBI blastp search identified the closest homologous match for Et_7A_051114 in Heller's rosette grass [*Dichanthelium oligosanthes* (Schult.)], encoding the GTP-binding protein YPTM1 (OEL32215). YPTM1 belongs to the Rab family of small GTPases, which play essential roles in vesicle transport and membrane trafficking in plants (Bischoff *et al*. 1999). GO enrichment analysis revealed 3 significant biological processes related to this gene: pollen tube development, lipoprotein metabolism, and endoplasmic reticulum-to-Golgi vesicle-mediated transport (FDR; *P* < 0.05).

Several genes identified through RDA were located near the iHH12 sweep regions. Notably, 1 locus on chromosome 2A at 33,374,745 bp was detected ∼45 kb from the nearest sweep region. This locus was predicted to cause a nonsynonymous substitution within the coding sequence of Et_2A_015154: c.702C > T (p.Asp235Asn). The RDA model showed a positive correlation between this locus and temperature annual range (bio7; *r* = 0.237), with the alternate allele associated with increased annual temperature variability (Fig. 3). Although 3 additional RDA candidates were identified within the coding regions on chromosome 2A, none of these loci altered their protein sequences. We conducted a one-way ANOVA to evaluate the effects of environmental and spatial factors on Et_2A_015154, which revealed significant associations between temperature annual range (bio7), latitude, and its allelic state (*P* ≤ 0.05). An NCBI blastp search identified Et_2A_015154 as homologous to mitogen-activated protein kinase (MAPK) kinase kinase 1 in green-millet (XP_034591805), a member of the MAPK cascade involved in regulating diverse cellular processes and stress responses (Colcombet and Hirt 2008; Rodriguez *et al*. 2010; Sinha *et al*. 2011; Šamajová *et al*. 2013; Smékalová *et al*. 2014; Liu *et al*. 2015, 2019; Vadovič *et al*. 2019; Li *et al*. 2021). Further analysis revealed a significant enrichment of GO terms related to kinase regulation and phosphorylation (FDR; *P* < 0.05).

### Future climate scenarios reveal maladaptation risks for traditional tef varieties in northern Ethiopia

**Fig. 5.**
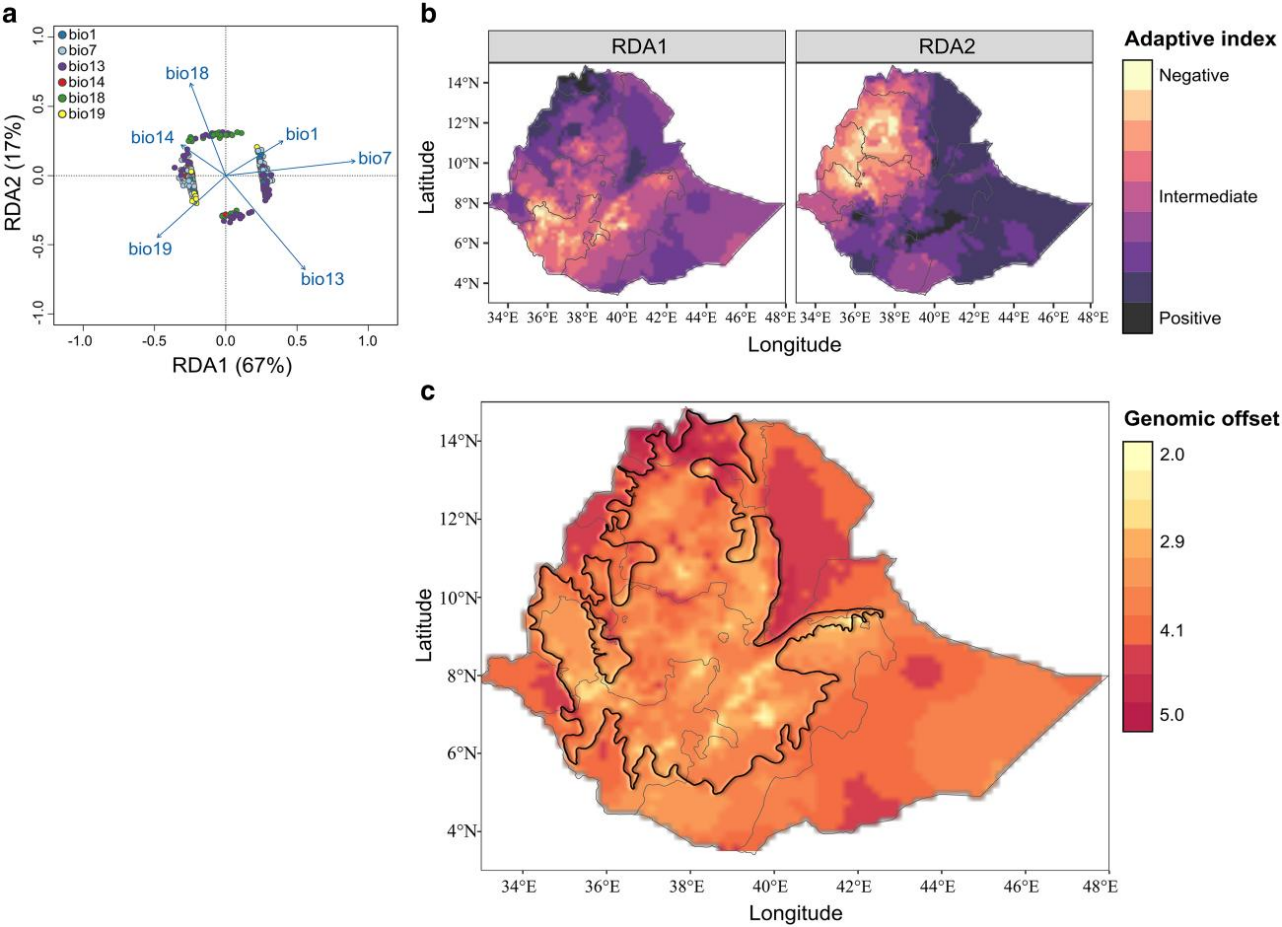
Current and predicted adaptive landscape of *E. tef* in Ethiopia. a) Biplot projection of 482 significant RDA loci and 6 bioclimatic variables into the adaptively enriched genetic space. b) Spatial extrapolation of RDA1 and RDA2 adaptive genetic gradients under current climatic conditions. c) Average predicted genomic offset by the year 2080, estimated using the SSP2-4.5 climate scenario and the multimodel ensemble projection of 12 CMIP6 Global Circulation Models (refer to Supplementary Table 2 for model descriptions). The bolded black outline within the country perimeters highlights a region of current land suitability for *E. tef*, based on the spatial projection of the landscape metadata collected by and provided through the EIAR.

The “adaptively enriched genetic space” reveals the strong correlations between genetic markers and diverse bioclimatic variables, as well as the interrelationships among these variables (Fig. 5a). Notably, most of the observed variation was aggregated on RDA1 (67% of the variance), to which allele frequencies were associated with either high temperature annual range (bio7) and high precipitation of the wettest month (bio13) (positive RDA1 scores) or low precipitation in the coldest quarter (bio19) (negative RDA1 scores). This adaptive gradient, when projected onto the landscape, delineates the contrasting conditions between low and high elevation areas, as indicated by the positive and negative RDA1 scores (Fig. 5b). Additionally, the adaptive gradients related to RDA2 (17% of the variance) distinguished 2 distinct regions (i.e. Amhara and Benshangul-Gumaz) on the landscape. These regions exhibited lower precipitation levels, particularly during the colder months (negative RDA2 scores), whereas other regions were characterized by higher precipitation levels, especially during the warmer months (positive RDA2 scores). To better understand the potential risks of future maladaptation, we used our enriched RDA model to predict the optimal adaptive indices for current and future environmental conditions across Ethiopia. We estimated shifts between these 2 predictions (i.e. genomic offset), predicting a contrasting pattern for 2080 (Fig. 5c). Our model predicted a risk of maladaptation in northern Ethiopia (genomic offset ≥ 4) compared with the rest of the region currently suitable for tef cultivation.

## Discussion

In this study, we conducted a large-scale genome–environment association analysis of a previously unexamined, diverse collection of Ethiopian tef. We employed whole-genome sequencing to test the hypothesis that traditional varieties of tef are locally adapted along abiotic environmental clines throughout its range of cultivation. Our analysis revealed that geographic distance had a minimal influence on the observed genetic structure. Through a combination of multivariate genotype–environment association methods and genome-wide selection scans, we identified genetic variations exhibiting signatures of both diversifying and positive selection. Finally, we used these results to predict the spatial distribution of adaptive genetic variation across Ethiopia to highlight regions potentially at risk of maladaptation under future climatic conditions.

Geographic distance often limits gene flow, and combined with local genetic drift, it leads to spatially structured differences in allele frequencies, resulting in a pattern of IBD (Wright 1943; Charlesworth *et al*. 2003). Traditional tef varieties were sampled and resequenced from across 5 regions (Amhara, Tigray, Benishangul-Gumuz, Oromia, and the Southern Nations Nationalities and Peoples). Spatial and environmental autocorrelation analyses showed no evidence of IBD or IBE, as ${\bar{F}}_{ST}$ values did not strongly correlate with geographic distance (Mantel *r* = −0.006, *P* = 0.505) or environmental distance (Mantel *r* = 0.162, *P* = 0.071) (Fig. 1). These findings were consistent with previous studies documenting the extensive seed trade and genetic admixture within informal seed systems among Ethiopian smallholder farmers (Negassa 1985; Ferede 2013; Abebe *et al*. 2013; Girma *et al*. 2018). Although sociocultural factors, such as kinship systems and ethnolinguistic groups (Samberg *et al*. 2013; Labeyrie *et al*. 2016), and environmental factors, such as elevation (Woldeyohannes *et al*. 2020), have been identified as influential in seed-mediated gene flow, our data does not allow us to separate the effects of spatial and environmental from social intervention. Woldeyohannes *et al*. (2020) showed that smallholder farmers consistently preferred the same tef ideotype regardless of gender, genetic background, or locality, favoring high-yielding, high-biomass, and fast-maturing varieties. Further research is warranted to achieve a comprehensive understanding of the role of social processes in shaping spatial patterns of genetic diversity in tef.

Partial RDA-based variance revealed that only a small proportion of genetic variation (0.18%) was jointly explained by climate and population structure (Fig. 2a; Table 1). This approach has been widely applied to estimate the relative contributions of climate and space explaining the genetic variation in species such as Arabidopsis (Lasky *et al*. 2012), *Eucalyptus globulus* (Labill.) (Butler *et al*. 2022), and barley (Abebe *et al*. 2015). While correcting for population structure can reduce false positives when a neutral genetic structure follows selective gradients, it may also eliminate true signals, leading to false negatives (Excoffier *et al*. 2009). Forester *et al*. (2018) demonstrated that RDA maintains low false positive rates and high statistical power for detecting selective events, with only a slight increase in false positives when accounting for population structure. These findings were consistent across demographic histories, sampling designs, and levels of population structure. Given the weak spatial genetic structure and lack of shared genetic variance between population structure and climate in our dataset, we chose not to apply population structure-based corrections in the RDA model for downstream genotype–environment associations (Fig. 1c; Table 1).

Climate variables in the RDA model accounted for ∼0.63% of the genetic variation, identifying 482 climate-associated RDA loci, with temperature annual range (bio7) and precipitation of the wettest month (bio13) as the main climate drivers of the detected genetic variation (Fig. 2). Although the observed proportion of genetic variation explained by climate variables was relatively low, it aligns with expectations under the neutral theory of molecular evolution (Kimura 1983). Therefore, only a small subset of loci are expected to show significant relationships with environmental variables. Among these loci, 145 (30.1%) were identified near or within the *E. tef* v3 coding sequence regions, with 32 (6.6%) conferring nonsynonymous sequence changes. Enrichment analysis of these candidate regions identified 5 distinct functional categories with 31 enriched biological process GO terms that were associated with 92 genes (FDR; *P* < 0.05). These candidate genes represent promising targets for understanding climate-driven adaptation. However, their interpretation warrants caution due to potential limitations in sample size and the underlying genetic structure of tef, a predominantly self-pollinating species with low rates of outcrossing (0.1–1%) (Berhe *et al*. 2000; Charlesworth *et al*. 2003).

GO terms related to stress responses and protein modification were strongly correlated with precipitation of the wettest month (bio13) and temperature annual range (bio7) (Supplementary Fig. 4). Et_2A_015154, encoding a homologue of the MAPK protein family (MAPK kinase kinase 1), exhibited significant positive correlations with temperature annual range (bio7) and latitude (*P* < 0.05). The alternate allele frequency of this locus was notably higher in high-latitude regions (12°–14°N) characterized by greater temperature variability (Fig. 3). MAPKs play crucial roles in plant responses to abiotic stress, including cold, salt, heat, and UV radiation, serving as convergence points for transmitting environmental stress signals to downstream targets (Sinha *et al*. 2011). These pathways are well-documented in several plant species, such as Arabidopsis, rice, tomato [*Lycopersicon esculentum* (Miller.)], and cotton [*Gossypium hirsutum* (L.)], underscoring their role in facilitating adaptation to environmental stressors.

The distribution of adaptive genetic variation across a landscape shapes how species respond to climate change (Jump and Peñuelas 2005; Aitken *et al*. 2008; Steane *et al*. 2014; Capblancq *et al*. 2020; Capblancq and Forester 2021). As the climate shifts, populations adapted to current local climates may struggle to achieve optimal fitness levels, creating a mismatch between existing genotypes and the new environment (Aitken *et al*. 2008; Capblancq *et al*. 2020). We estimated the adaptive genomic offset between present and projected climates, identifying northern Ethiopia, particularly the Tigrayan region, as having the highest predicted risk of maladaptation (genomic offset ≥ 4). In this region, allele frequencies were strongly associated with a higher temperature annual range (bio7) and high precipitation of the wettest month (bio13) (Fig. 5). However, it is important to acknowledge that these tests provide indirect measures of fitness, and the assumption of linearity in RDA may overlook potential nonlinear relationships between the tested environmental gradients and associated genetic variation. Projected climate changes under CMIP6 SSP2-4.5 scenarios for 2061–2080 suggest that East Africa will face intensified seasonal rainfall variability, which may further exacerbate agricultural challenges. In Tigray, prolonged exposure to drought has historically driven the selection of earlier maturation and shorter panicle architectures to sustain grain yield (Woldeyohannes *et al*. 2020). Further research is needed to identify and evaluate genetic backgrounds with enhanced environmental resilience suited to the projected climates of regions more vulnerable to climate change in tef cultivation.

Two additional outlier detection approaches were applied to examine environment-driven genetic differentiation (${\bar{F}}_{ST}$) and intraspecific selective events (iHH12). These analyses identified selection signatures in 291 distinct genomic regions spanning 20 chromosomes. The genome scans using iHH12 statistics showed the strongest enrichment for recent selective sweeps on chromosomes 1A, 1B, and 2. In contrast, ${\bar{F}}_{ST}$ analysis identified 4 regions of significant genetic divergence on chromosomes 2A, 5A, and 7A, which exhibited a shared genetic response to annual mean temperature (bio1), precipitation of the warmest quarter (bio18), and altitude (Supplementary Table 3 and Fig. 3). Notably, no candidate regions were consistently identified across all approaches, reflecting the differing selection signals detected by these methods. This is illustrated in Fig. 3, where an inverse relationship was observed between putative sweep regions (high iHH12 scores) and environment-driven divergent alleles (high ${\bar{F}}_{ST}$ values). This pattern aligns with local adaptation processes, where allele frequencies are maintained at intermediate levels, in contrast to the high-frequency haplotypes with extended LD indicated by elevated iHH12 scores (Price *et al*. 2018). Similarly, RDA loci showed a comparable relationship with iHH12 sweep regions, suggesting that these regions associated with climate were subject to distinct selective pressures, likely unrelated to recent selective events within the population.

Tef, along with other orphan crops, plays a key role in the subsistence of local communities and presents promising solutions to food insecurity and climate change vulnerability. Despite these advantages, orphan crops, like tef, have lagged behind major crops in terms of improvement efforts. This has largely been due to limited research investment and limited selective pressures by subsistence farmers, resulting in undesirable agronomic traits reminiscent of wild relatives (Lemmon *et al*. 2018). To overcome these challenges, research focusing on the characterization of larger and more diverse germplasm collections, coupled with deeper genome sequencing efforts, is essential to identify the genetics underlying environmental adaptation in tef. Controlled experiments, such as common gardens and in silico analyses, will be critical for functionally validating climate-driven candidate genes. Specifically, we propose developing and testing near-isogenic lines or genetically engineered lines that isolate specific variants to isolate specific variants and empirically test their effects on plant performance and fitness across diverse environments. With 95% of Ethiopia's crop area reliant on rainfed agriculture, limited water availability and rising temperatures pose significant challenges to tef cultivation, particularly in lowland regions (International Trade Administration 2024). Collaborative efforts that leverage the genetic diversity of indigenous tef germplasm, combined with advanced genomic technologies, present valuable opportunities for characterizing and utilizing locally adaptive genetic variation to support the sustainable intensification of agricultural systems under climate change (Assefa *et al*. 2011; Beyene *et al*. 2022).

## Supplementary Material

jkae303_Supplementary_Data

## Data Availability

Raw DNA sequencing reads are published to the National Center for Biotechnology Information (NCBI) Short Read Archive (https://www.ncbi.nlm.nih.gov/sra/) under BioProject ID PRJNA1112804. Scripts used for data analysis are available on GitHub at (https://github.com/gvp681/tef_landscape_genomics) and the accompanying metadata can be found on the Dryad Digital Repository (doi:10.5061/dryad.8kprr4xw3). Supplemental material available at G3 online.
