# Supplementary material for: Genetic diversity and environmental adaptation in Ethiopian tef
Source: G3 (Bethesda). 2025 Jan 24;15(3):jkae303. doi: 10.1093/g3journal/jkae303 (PMC11917483; doi:10.1093/g3journal/jkae303)
Supplement: jkae303_Supplementary_Data [file jkae303_supplementary_data.zip › Supplemental_Material_G3-2024-405448.pdf]

Genetic Diversity and Environmental Adaptation in Ethiopian Tef

Kirsten M. Hein, Dejene Girma, and John K. McKay

Supplemental Figures (Pages 2-4)

Supplemental Figure S1.
Supplemental Figure S2
Supplemental Figure S3
Supplemental Figure S4

Supplemental Tables (Pages 5-6)

Supplemental Table S1.
Supplemental Table S2
Supplemental Table S3

Supplemental File S1 (Additional_Files_SuppInfo.xlsx)

- **S1.1**: Detailed information on tef germplasm collection, DNA extraction, and plate coordinates referenced through the Illumina NovaSeq 6000 platform.
- **S1.2**: Geographic and environmental (WorldClim version 2.1) data associated with each sample.
- **S1.3**: Individual population genetic assignments for all samples, including axis loadings from discriminant analysis of principal components (LD1 - LD4), principal components analysis (PC1 and PC2), and K-means cluster assignments.
- **S1.4**: Genetic distances, estimated as pairwise FST, alongside geographical and environmental distance matrices, based on 39 individual samples from 16 distinct geographic locations and using 20 WorldClim environmental variables. Only sampling locations with two or more collected varieties were included.
- **S1.5**: Redundancy analysis model output (*without correction for population structure*), including identified outlier loci and correlation coefficients to each environmental variable..
- **S1.6**: Redundancy analysis model output (*with correction for population structure*), including identified outlier loci and correlation coefficients to each environmental variable.
- **S1.7**: Detailed genotype information and associated metadata for each non-synonymous site identified by the redundancy analysis.
- **S1.8**: Genome-wide sliding window iHH12 scores calculated using Selscan v2.0.0 (Szpiech, 2021; Szpiech & Hernandez, 2014).
- **S1.9**: Genome-wide sliding window $\overline{F}_{ST}$ values calculated using VCFtools version 0.1.16 (Danecek et al., 2011; Weir & Cockerham, 1984).
- **S1.10**: GO enrichment analysis results, including GO terms and descriptions of biological processes, for *E. tef* v3 genes associated with outlier loci identified through redundancy analysis.
- **S1.11**: GO enrichment analysis results, including GO terms and descriptions of biological processes, and their associated environmental variables from redundancy analysis.

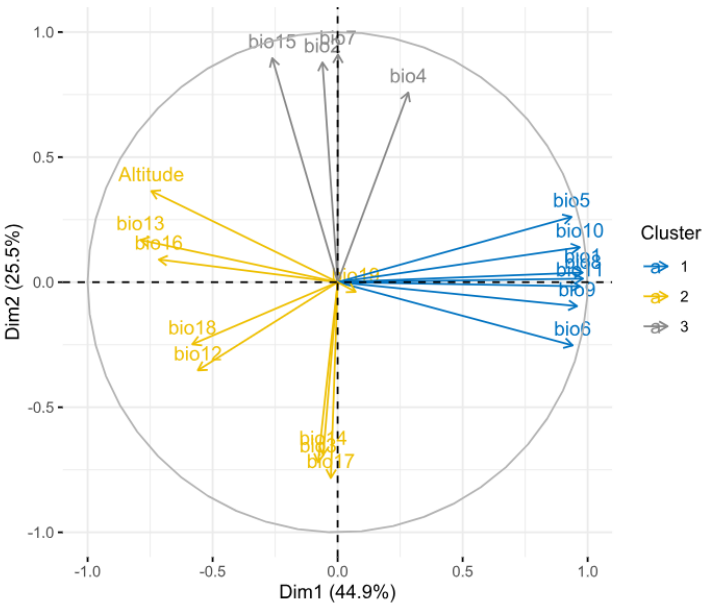

Figure S1. PCA biplot of environmental variables in Ethiopia, encompassing 19 bioclimatic variables and altitude. Loading vectors are grouped using k-means clustering (K = 3) to visualize similarities among variables.

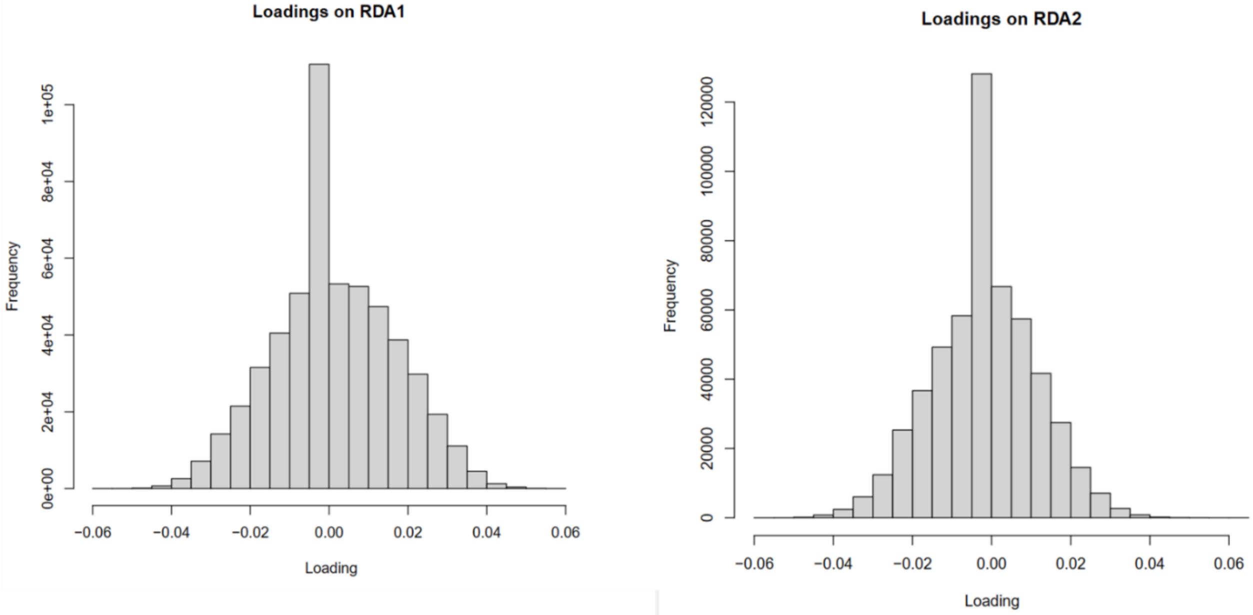

Figure S2. Distribution of locus loadings on the first two RDA axes. The position of loci within each distribution reflects potential relationships with environmental predictors, with loci positioned in the center indicating weaker environmental associations and at the tails suggesting stronger environmental associations.

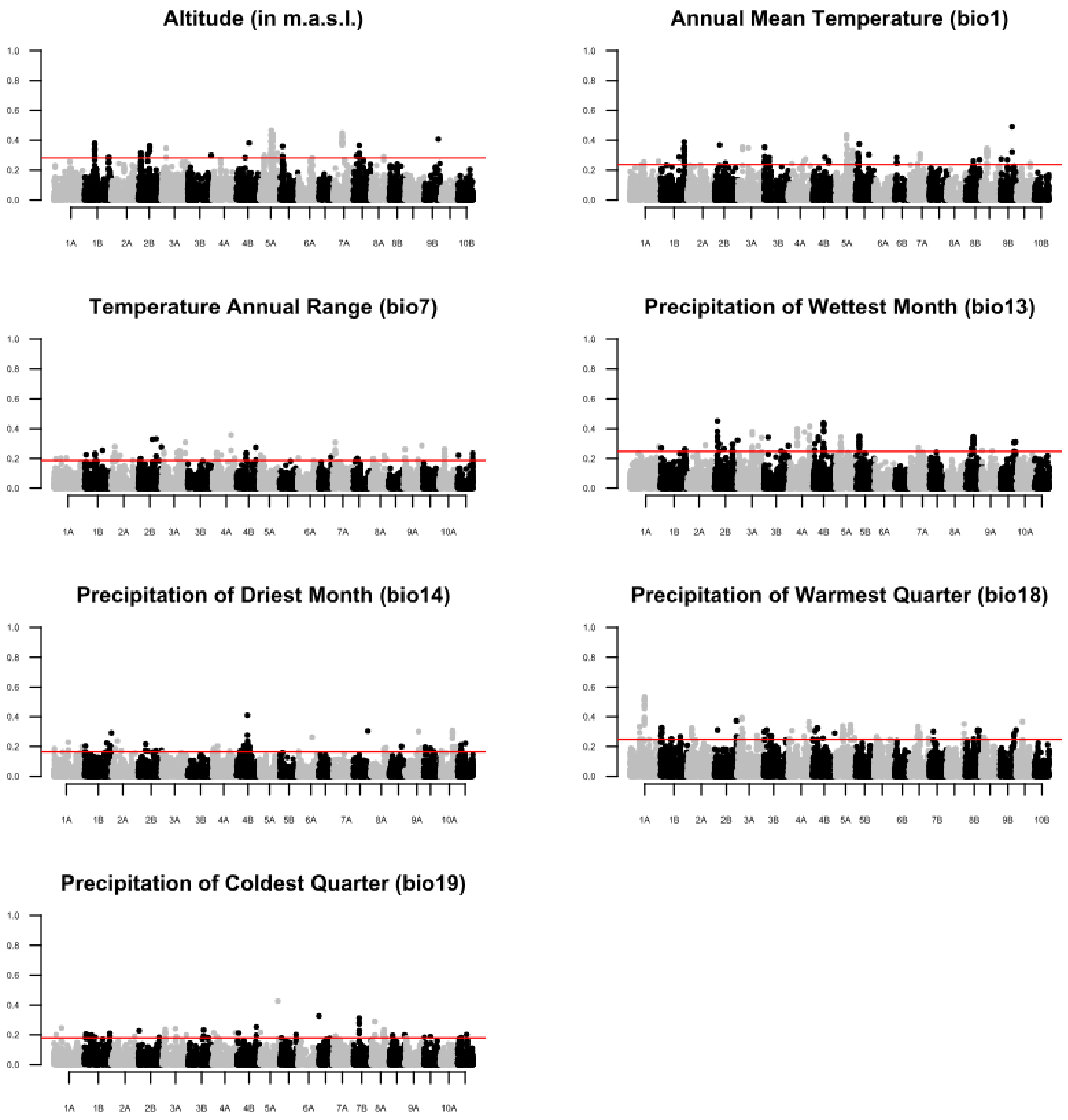

Figure S3. Manhattan plots of genome-wide $\overline{F}_{ST}$ across 20-kb sliding windows between populations under divergent environmental conditions. Genetic markers are positioned along the 20 chromosomes for each of six bioclimatic variables and altitude. The red line indicates the top 1% threshold for $\overline{F}_{ST}$ for each environmental variable.

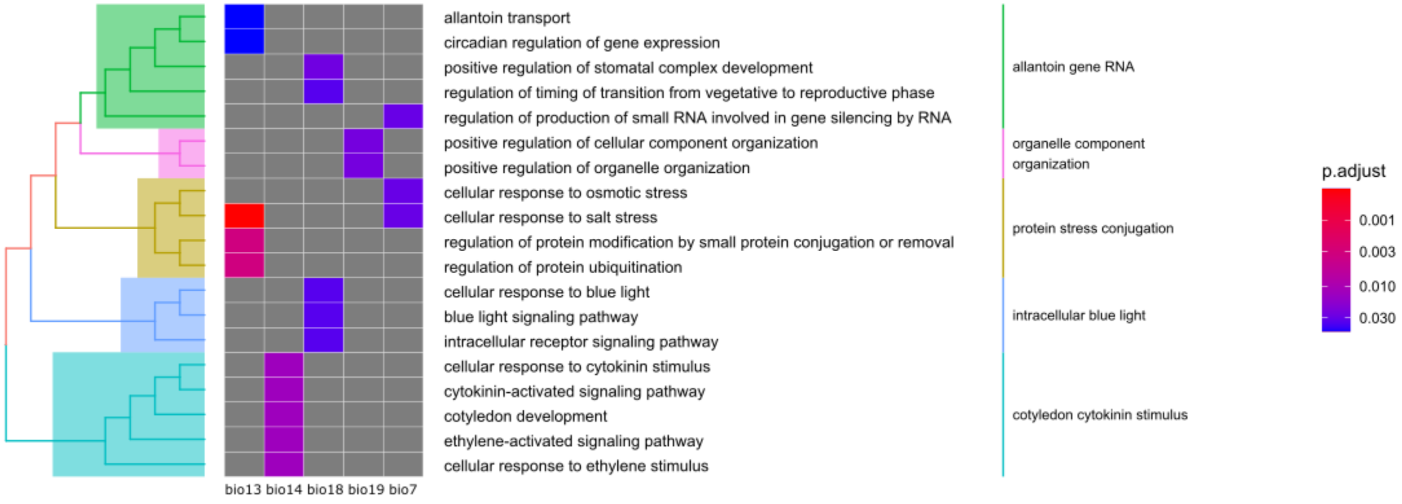

Figure S4. Functional grouping tree diagram for GO enrichment result from gene set enrichment analysis. Red and blue represent smaller and larger p-values, respectively. The analysis involved 145 loci, revealing 92 genes (FDR $p < 0.05$), across five bioclimatic variables gene IDs. Precipitation of the wettest month (bio13), precipitation of the driest month (bio14), precipitation of the warmest quarter (bio18), precipitation of the coldest quarter (bio19), and temperature annual range (bio7), were enriched with 23, 1, 5, 12, and 51 genes, respectively. The top 19 enriched GO terms revealed five distinct functional categories involving 38 genes.

Table S1. Description of WorldClim version 2.1 climate and altitudinal variables (www.worldclim.org).

Predictor	Description
bio1	Annual Mean Temperature
bio2	Mean Diurnal Range (Mean of monthly (max – min temperature))
bio3	Isothermality (BIO2/BIO7) (×100)
bio4	Temperature Seasonality (standard deviation ×100)
bio5	Max Temperature of Warmest Month
bio6	Min Temperature of Coldest Month
bio7	Temperature Annual Range (BIO5-BIO6)
bio8	Mean Temperature of Wettest Quarter
bio9	Mean Temperature of Driest Quarter
bio10	Mean Temperature of Warmest Quarter
bio11	Mean Temperature of Coldest Quarter
bio12	Annual Precipitation
bio13	Precipitation of Wettest Month
bio14	Precipitation of Driest Month
bio15	Precipitation Seasonality (Coefficient of Variation)
bio16	Precipitation of Wettest Quarter
bio17	Precipitation of Driest Quarter
bio18	Precipitation of Warmest Quarter
bio19	Precipitation of Coldest Quarter
Altitude	Altitude (in m.a.s.l.)

Table S2. CMIP6 Global Circulation Models utilized for estimation of genomic offset for *E. tef* in Ethiopia.

Model Name	Source
ACCESS-CM2	(Dix et al., 2019)
CMCC-ESM2	(Lovato et al., 2023)
EC-Earth3-Veg	(EC-Earth Consortium (EC-Earth), 2019)
FIO-ESM-2-0	(Song et al., 2019)
GISS-E2-1-G	(NASA Goddard Institute for Space Studies (NASA/GISS), 2020)
HadGEM3-GC31-LL	(Good, 2019)
INM-CM5-0	(Volodin et al., 2019)
IPSL-CM6A-LR	(Boucher et al., 2019)
MIROC6	(Shiogama et al., 2019)
MPI-ESM1-2-HR	(Schupfner et al., 2019)
MRI-ESM2-0	(Yukimoto et al., 2019)
UKESM1-0-LL	(Good et al., 2019; Shim et al., 2020)

Table S3 Summary of 20-kb genomic regions exhibiting the top 1% elevated $\overline{F}_{ST}$ values and associated environmental variables.

Chromosome	Start Position (bp)	End Position (bp)	Environment
Chr1A	18,880,001	19,140,001	bio18
Chr1B	1,420,001	1,460,000	bio18
Chr1B	13,320,001	13,560,000	altitude
Chr1B	31,000,001	31,180,000	bio1
Chr2A	5,180,001	5,380,000	bio18
Chr2B	3,800,001	3,920,000	bio13, altitude
Chr2B	15,000,001	15,060,000	altitude
Chr2B	23,500,001	23,680,000	bio7, bio13
Chr2B	29,120,001	29,260,000	bio18
Chr3A	5,600,001	6,100,000	bio1, bio18, altitude
Chr3A	19,240,001	19,280,000	bio13
Chr3B	1,140,001	1,220,000	bio1
Chr4A	11,260,001	11,380,000	bio13
Chr4A	27,760,001	27,800,000	bio13
Chr4B	6,140,001	6,740,000	bio18
Chr4B	13,600,001	13,940,000	bio13
Chr5A	6,460,001	6,500,000	bio13, altitude
Chr5A	9,960,001	10,100,000	bio18
Chr5A	1,502,000	17,500,000	bio1, altitude
Chr5A	20,140,001	20,260,000	bio18
Chr5A	23,100,001	23,410,000	bio1, altitude
Chr5B	3,280,001	3,400,000	bio1, altitude
Chr5B	3,980,001	4,200,000	bio1, bio13
Chr7A	3,320,001	3,360,000	bio7
Chr7A	11,400,001	12,380,000	bio18, altitude
Chr7A	14,260,001	14,500,000	bio1, bio18, altitude
Chr7B	7,820,001	8,080,000	bio19, altitude
Chr8A	22,480,001	22,560,000	bio18
Chr8B	10,420,000	10,640,000	bio13
Chr9A	3,760,001	4,000,000	bio18
Chr9A	7,200,001	7,460,000	bio1, bio18
Chr10A	16,660,001	16,920,000	bio14
